# Causal relationship between gut microbiota and myasthenia gravis: a bidirectional mendelian randomization study

**DOI:** 10.1186/s13578-023-01163-8

**Published:** 2023-11-07

**Authors:** Tengfei Su, Xiang Yin, Jiaxin Ren, Yue Lang, Weiguanliu Zhang, Li Cui

**Affiliations:** Department of Neurology, the First Hospital of Jilin University, Changchun, China

**Keywords:** Gut microbiota, Myasthenia gravis, Short-chain fatty acids, Causal analysis, Foxp3 + CD4 + Treg cells

## Abstract

**Background:**

Observational studies have demonstrated an association between gut microbiota and myasthenia gravis; however, the causal relationship between the two still lacks clarity. Our goals are to ascertain the existence of a bidirectional causal relationship between gut microbiota composition and myasthenia gravis, and to investigate how gut microbiota plays a role in reducing the risk of myasthenia gravis.

**Methods:**

We acquired gut microbiota data at the phylum, class, order, family, and genus levels from the MiBioGen consortium (N = 18,340) and myasthenia gravis data from the FinnGen Research Project (426 cases and 373,848 controls). In the two-sample Mendelian randomization analysis, we assessed the causal relationship between the gut microbiota and myasthenia gravis. We also conducted bidirectional MR analysis to determine the direction of causality. The inverse variance weighted, mendelian randomization-Egger, weighted median, simple mode, and weighted mode were used to test the causal relationship between the gut microbiota and severe myasthenia gravis. We used MR-Egger intercept and Cochran's Q test to assess for pleiotropy and heterogeneity, respectively. Furthermore, we utilized the MR-PRESSO method to evaluate horizontal pleiotropy and detect outliers.

**Results:**

In the forward analysis, the inverse-variance weighted method revealed that there is a positive correlation between the genus Lachnoclostridium (OR = 2.431,95%CI 1.047–5.647, p = 0.039) and the risk of myasthenia gravis. Additionally, the family Clostridiaceae1 (OR = 0.424,95%CI 0.202–0.889, p = 0.023), family Defluviitaleaceae (OR = 0.537,95%CI  0.290–0.995, p = 0.048), family Enterobacteriaceae (OR = 0.341,95%CI  0.135–0.865, p = 0.023), and an unknown genus (OR = 0.407,95%CI  0.209–0.793, p = 0.008) all demonstrated negative correlation with the risk of developing myasthenia gravis. Futhermore, reversed Mendelian randomization analysis proved a negative correlation between the risk of myasthenia gravis and genus Barnesiella (OR = 0.945,95%CI  0.906–0.985, p = 0.008).

**Conclusion:**

Our research yielded evidence of a causality connection in both directions between gut microbiota and myasthenia gravis. We identified specific types of microbes associated with myasthenia gravis, which offers a fresh window into the pathogenesis of this disease and the possibility of developing treatment strategies. Nonetheless, more studies, both basic and clinical, are necessary to elucidate the precise role and therapeutic potential of the gut microbiota in the pathogenesis of myasthenia gravis.

**Supplementary Information:**

The online version contains supplementary material available at 10.1186/s13578-023-01163-8.

## Introduction

Myasthenia gravis (MG) is an autoimmune disease characterized by the attack of antibodies on the neuromuscular junction, leading to muscle weakness. The most common symptom is ocular muscle weakness, which can also affect bulbar, limb, axial, and ventilator muscles, progressing to generalized MG [1]. Acute respiratory failure requiring mechanical ventilation occurs in 20% of patients with MG in clinical practice, resulting in significantly increased mortality [2]. The etiology of MG is multifaceted, resulting from genetic and several environmental risk factors [3–5]. However, the exact factors that result in an individual's susceptibility to MG are unknown. The gut microbiota is critical for the development and maintenance of host metabolism and immune homeostasis in the human intestine, which affects human nutrition as well as gastrointestinal function and integrity [6, 7]. Mounting scientific evidence indicates that microbial-host interactions affect not only the gut environment but also remote organs [8, 9]. Evidence suggests that gut microbiota can significantly affect both the innate and adaptive immune systems and have a dual role in either promoting or protecting against disease development [10]. Studies suggest that disturbances in the composition of the gut microbiota can be associated with several autoimmune diseases, including systemic lupus erythematosus, rheumatoid arthritis and multiple sclerosis [11].

Alterations in the composition of the gut microbiota have been observed in both myasthenia gravis patients and animal models. A case–control study demonstrated that the gut microbiota diversity and abundance differed between the myasthenia gravis (MG) group and the healthy control group. Specifically, the MG group exhibited reduced levels of Firmicutes, Clostridium, Eubacterium, and F. prausnitzii. Conversely, higher levels of Proteobacteria, Bacteroidetes, Streptococcus, and Parasutterella were noted in the MG group. The healthy control group displayed Clostridium levels approximately three times that of the MG group [12]. Based on German Moris et al.'s research, it was found that Verrucomicrobiaceae and Bifidobacteriaceae were less abundant in patients with MG than in the healthy control group, while the abundance of Bacteroidetes and Desulfovibrionaceae was greater [1]. An animal experiment showed that transplanting microbiota from MG mice to germ-free mice resulted in reduced motor function, which could be restored through a mixed microbiota (made up of both the MG and healthy microbiota) [2]. This suggests that the gut microbiota may play a role in MG development.

However, observational studies dominate most current research; while suggesting an association between gut microbiota and MG, the conclusions drawn in observational studies tend to be based on “association” rather than “causation”. Additionally, confounding factors like demographics, comorbidities, medications, and diet cannot be ruled out. And, experiments conducted on animals do not guarantee the same results in humans. The Mendelian Randomization method aims to alleviate these limitations.

Mendelian randomization (MR) is a method of data analysis widely used recently to infer causality in epidemiology. Traditional epidemiological causal inference is hampered by reverse causation and confounding factors. Randomized controlled trials are difficult to implement due to ethical limitations in human medicine and trial design. MR complements these limitations by using gene variation as instrumental variables (IVs), normally single nucleotide polymorphisms (SNPs), to infer the causal effect of treatment exposure on outcomes [13]. Mendel’s laws of inheritance ensure a random distribution of parental alleles to offspring during gamete formation, thereby minimizing interference from common confounding factors such as postnatal environment, socioeconomic status, and behavioral factors [13, 14]. This reasonable causal sequence results in a more accurate estimation of true causal effects. The present study aims to shed light on the association between changes in gut microbiota and MG using MR method.

## Materials and methods

### Study design

**Fig. 1:**
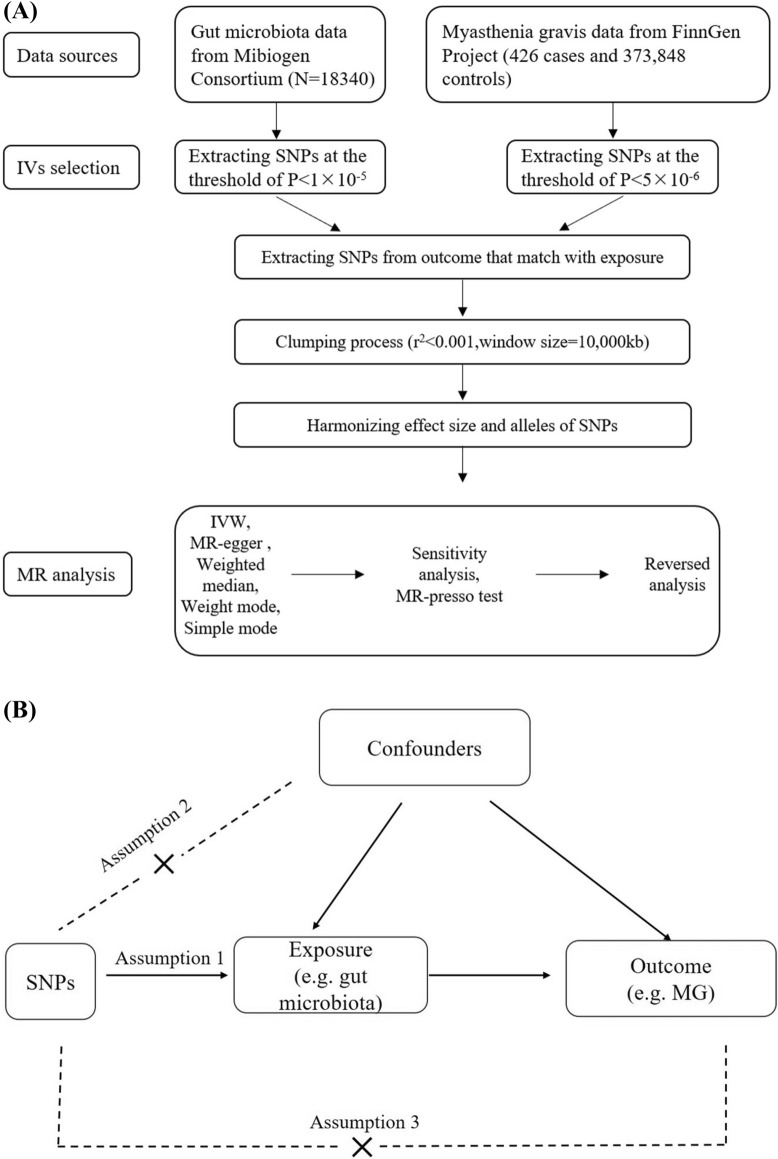
Study design and Mendelian randomization core assumption. **A** Data sources and study design of bidirectional Mendelian randomization. **B** Three core assumptions in the Mendelian randomization

We conducted a bidirectional MR analysis to investigate the causal relationship between the gut microbiota and MG; the flowchart and design for this analysis is presented in Fig. 1A. First, the genome-wide association study (GWAS) data for the gut microbiota and MG were obtained from the Mibiogen Consortium [15] and FinnGen Research Project, respectively. Genetic variants from the GWAS data were retrieved and used as IVs. Then, a Two-Sample MR was performed using the R package “TwoSampleMR” (0.5.6), which included five MR methods. Sensitivity analyses, including pleiotropy [16] and heterogeneity tests [17], and leave-one-out analyses were conducted.MR-PRESSO was used to detect and correct outliers [18]. Finally, reversed analysis was conducted to obtain a comprehensive conclusion regarding causation.

For results with minimal bias, adhering to three key assumptions is crucial in utilizing the MR method: (1) IVs were significantly correlated with exposure; (2) IVs did not have any confounding factors associated with exposure-outcome associations, and (3) IVs only affect outcomes through exposure [19] (Fig. 1B).

### Selection of data sources and instrumental variables

#### Data sources

The summary data on the gut microbiota utilized in this research was derived from a recently conducted genome-wide association study by the global consortium, MiBioGen [15]. The study’s vast database collected 16S ribosomal RNA gene sequencing and genotype data from 18,340 predominantly European participants (N = 13,266). The researchers amassed records of 211 bacterial characteristics belonging to 131 genera in nine phyla with sixteen classes, 20 orders, and 35 families. The data on MG was collected from the FinnGen Research Project, consisting of a group of 426 MG diagnosed cases and 373,848 European controls. The data used in this study was obtained from publicly available repositories and did not need any further ethics approval or patient consent.

### Instrumental variables selection

In the forward analysis, the exposure variable was gut microbiota, while the outcome variable was MG. The process for selecting IVs is illustrated in Fig. 1A.Firstly, a set of SNPs was selected as IVs which fell below the genome-wide significance threshold of 1 × 10^−5^ in line with previous studies[20]. Secondly, to ensure that each IV was independent, we clumped together SNPs utilizing the European 1000 Genomes Project reference dataset with an r^2^ value of 0.001 and a clumping window of 10,000 kb [21]. Third, we harmonized the effects of the SNP on outcome and exposure by ensuring that they referred to the same allele, correcting the strand for non-palindromic SNPs, and removing all palindromic sequences. The F-statistic calculated the potency of each SNP as an instrumental variable with a value exceeding 10 indicating a strong instrument.

During reverse causality studies with MG utilized as an exposure variable, a set of SNPs below the genome-wide threshold of statistical significance (5 × 10^−8^) was used as IVs; however, only a SNP reached this threshold. Thus, SNPs with a second *p*-value less than the genome-wide significance level (5 × 10^−6^) were selected to identify the underlying causal links. The remaining flow and parameters remained identical to the forward MR.

### Statistical analysis

To evaluate the causal link between gut microbiota and MG, multiple methods, including inverse variance weighted (IVW), MR-Egger regression, weighted median, simple mode, and weighted mode were employed. The primary analysis was conducted using the IVW method, while the other four methods supplemented the IVW [22]. The IVW method uses a meta-analytical approach to combine the Wald ratios for each SNP, and it provides the most precise estimates when all IVs are valid [23]. The MR-Egger regression relies on the “instrument strength independent of the direct effect” assumption that exposure and outcome are independent. It has lower precision and statistical power but can be used to correct for horizontal pleiotropy [16]. When up to 50% of the information originates from invalid genetic variants, the weighted median method provides the most unbiased estimate of the causal effects [24]. As an additional step, we used weighted mode and simple mode to enhance accuracy and stability [25].

Various sensitivity analyses were conducted to evaluate the strength of the results. MR-Egger intercept was employed to identify horizontal pleiotropy based on the distance of the regression intercept line from zero [26]. Cochran’s Q test is a method used to assess heterogeneity among different IVs. If the p-value of the Cochran's Q test is less than the pre-defined significance level (0.05), significant heterogeneity is considered to be present. Conversely, if the p-value is greater than the significance level (0.05), it is concluded that there is no significant heterogeneity among the IVs [27]. The leave-one-out sensitivity method was used to assess if one SNP significantly influenced causality estimates [28]. The MR-PRESSO test was used to detect and remove possible outliers, then provide estimates thereby correcting for horizontal pleiotropy [18]. Finally, the F-statistic for each SNP was computed using the formula β^2^/SE^2^ [28], with β and SE expressing the estimated and standard errors of the effect allele, respectively [29]. In this study, SNP with F-statistics less than or equal to 10 (defined as weak IVs) were excluded from the MR analysis. To adjust for multiple comparisons, we applied a Bonferroni correction, giving a cutoff of p = 2.37 × 10^−4^ (0.05/211) for gut microbiota and MG, and the corrected p > 0.05 was considered to suggest an association [30].

All statistical analyses were performed using R version 4.2.2. MR analyses were performed using the two-sample MR (version 0.5.6) [28] and MR-PRESSO (version 1.0)[31] R packages.

## Results

**Fig. 2:**
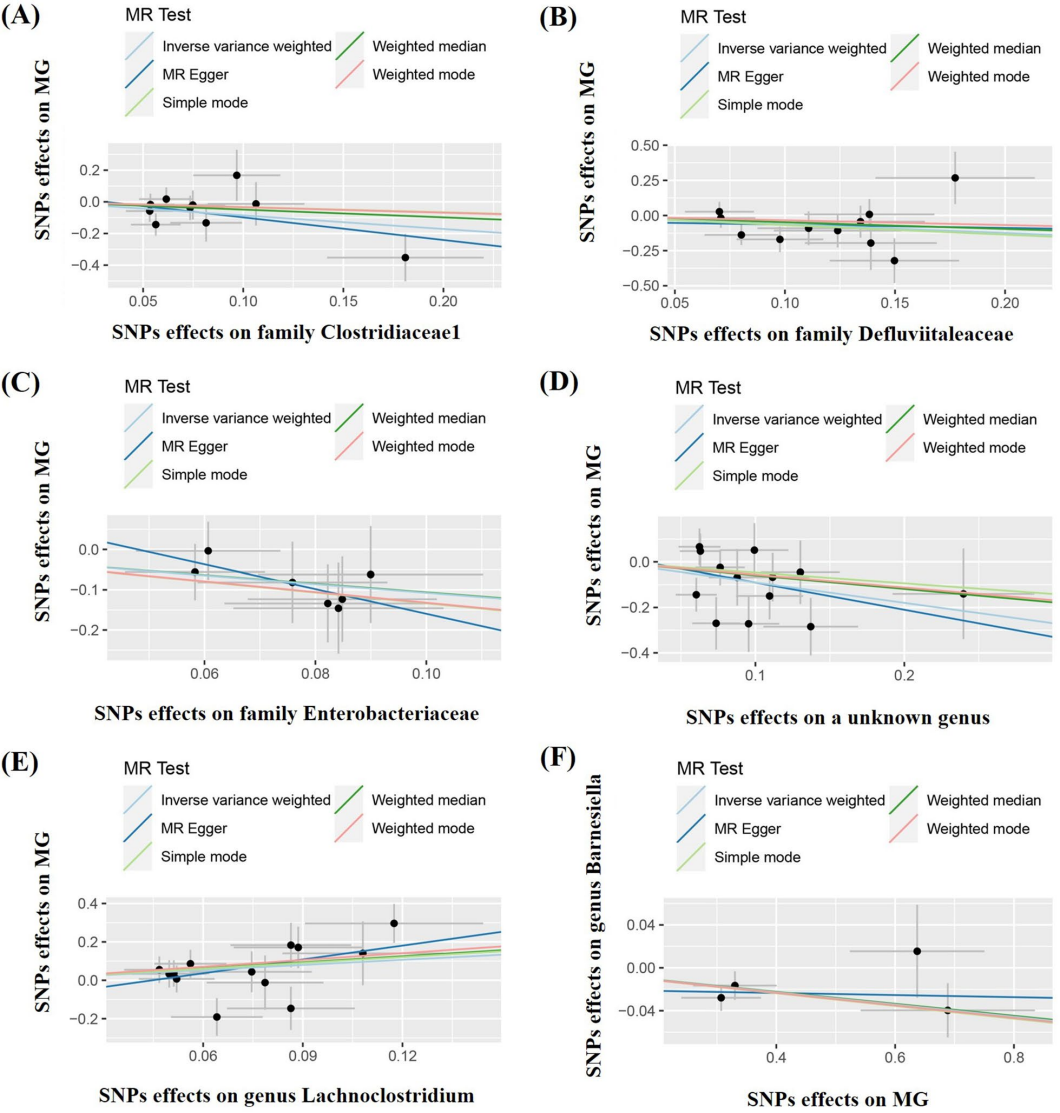
Scatter plots for the causal association between gut microbiota and MG

The results of IVW analyses demonstrated that the family Clostridiaceae1 (OR = 0.424,95%CI 0.202–0.889,p = 0.023), family Defluviitaleaceae (OR = 0.537,95%CI 0.290–0.995, p = 0.048), family Enterobacteriaceae (OR = 0.341,95%CI 0.135–0.865, p = 0.023), and an unknown genus (OR = 0.407,95%CI  0.209–0.793, p = 0.008) were negatively correlated with the risk of MG (Table 1 and Fig. 2A–D), whereas the genus Lachnoclostridium (OR = 2.431,95%CI  1.047–5.647, p = 0.039) was positively correlated with the risk of MG (Table 1 and Fig. 2E). The same results were obtained using the four additional methods.

**Table 1:** Results of the MR study testing causal association between genetically predicted gut microbiota and myasthenia gravis

Gut microbiota	Number of SNPs	Beta	P	OR (95% CI)	P for heterogeneity test	P for MR-Egger intercept	P for MR-PRESSO (0 outliers)
Family Clostridiaceae1							
IVW	10	− 0.857	0.023	0.424 (0.202–0.899)	0.528	0.595	0.600
MR Egger	10	− 1.429	0.230	0.239 (0.028–2.066)	0.458		
Weighted median	10	− 0.491	0.343	0.612 (0.222–1.689)			
Simple mode	10	− 0.328	0.721	0.720 (0.126–4.128)			
Weighted mode	10	− 0.344	0.681	0.709 (0.145–3.470)			
Family Defluviitaleaceae							
IVW	11	− 0.622	0.048	0.537 (0.290–0.995)	0.312	0.728	0.409
MR Egger	11	− 0.237	0.838	0.789 (0.087–7.139)	0.246		
Weighted median	11	− 0.479	0.244	0.619 (0.276–1.387)			
Simple mode	11	− 0.682	0.363	0.506 (0.125–2.053)			
Weighted mode	11	− 0.340	0.608	0.712 (0.202–2.059)			
Family Enterobacteriaceae							
IVW	7	− 1.076	0.023	0.341 (0.135–0.865)	0.968	0.508	0.737
MR Egger	7	− 3.068	0.329	0.047(0.000–12.120)	0.973		
Weighted median	7	− 1.063	0.081	0.345 (0.105–1.138)			
Simple mode	7	− 1.336	0.152	0.263 (0.053–1.296)			
Weighted mode	7	− 1.321	0.159	0.267 (0.053–1.335)			
Unknown genus							
IVW	13	− 0.899	0.008	0.407 (0.209–0.793)	0.242	0.759	0.174
MR Egger	13	− 1.199	0.263	0.302 (0.041–2.209)	0.189		
Weighted median	13	− 0.592	0.195	0.553 (0.226–1.335)			
Simple mode	13	− 0.469	0.523	0.626 (0.155–2.532)			
Weighted mode	13	− 0.561	0.389	0.571 (0.167–1.954)			
Genus Lachnoclostridium							
IVW	13	0.888	0.039	2.431 (1.047–5.647)	0.168	0.287	0.209
MR Egger	13	2.399	0.118	11.017 (0.688–176.305)	0.190		
Weighted median	13	1.043	0.042	2.838 (1.039–7.755)			
Simple mode	13	1.008	0.251	2.741 (0.533–14.092)			
Weighted mode	13	1.176	0.195	3.241 (0.604–17.402)			

*SNPs* single nucleotide polymorphisms, *OR* odds ratio, *IVW* inverse variance weighted

The summary data on gut microbiota is derived from a genome-wide association study involving 18,340 individuals. Data on myasthenia gravis were sourced from Finngen Research Project, comprising 426 cases and 373,848 controls

Reverse IVW analysis indicated that MG was associated with a lower abundance of the genus Barnesiella (OR = 0.945,95%CI 0.906–0.985, p = 0.008). The same results were obtained using the four additional methods (Table 2 and Fig. 2F).

**Table 2:** Results of the MR study testing causal association between genetically predicted myasthenia gravis and gut microbiota

Gut microbiota	Number of SNPs	Beta	P	OR (95% CI)	P for heterogeneity test	P for MR-Egger intercept	P for MR-PRESSO (0 outliers)
Genus Barnesiella							
IVW	4	− 0.057	0.008	0.945 (0.906–0.985)	0.537	0.526	0.660
MR Egger	4	− 0.010	0.895	0.990 (0.871–1.126)	0.450		
Weighted median	4	− 0.056	0.039	0.946 (0.897–0.997)			
Simple mode	4	− 0.059	0.189	0.942 (0.880–1.009)			
Weighted mode	4	− 0.058	0.152	0.943 (0.888–1.001)			

*SNPs* single nucleotide polymorphisms, *OR* odds ratio, *IVW* inverse variance weighted

The summary data on gut microbiota is derived from a genome-wide association study involving 18,340 individuals. Data on myasthenia gravis were sourced from Finngen Research Project, comprising 426 cases and 373,848 controls

**Fig. 3:**
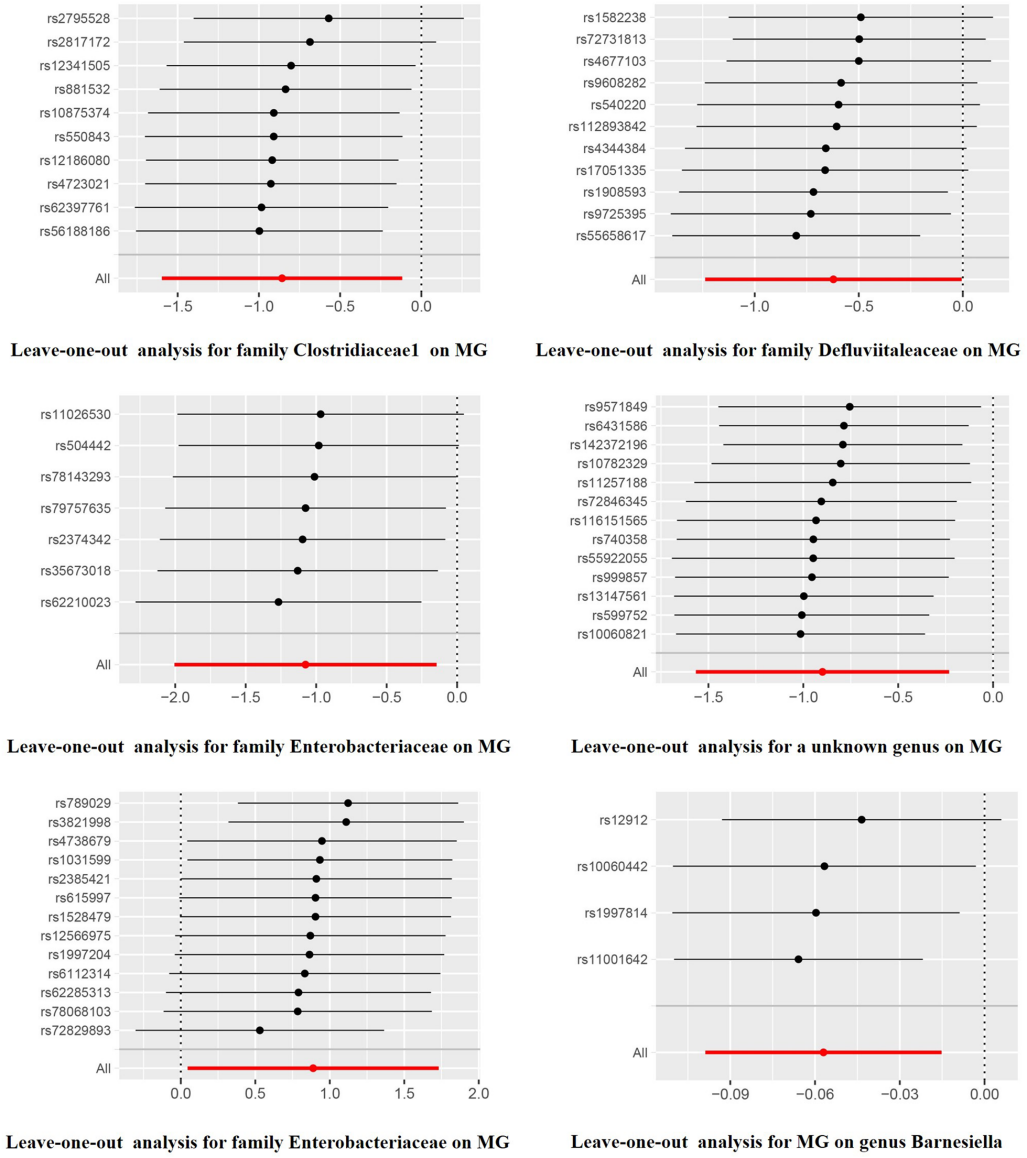
Leave-one-out analysis between gut microbiota and MG

No potential heterogeneity or pleiotropy was found in the sensitivity analysis (p > 0.05), as demonstrated in Table 1 and Table 2. Moreover, no notable outliers were identified in the MR-PRESSO or leave-one-out analyses (Table 1 and Figs. 3). The estimated F-statistics were all higher than 10, signifying that there were no weak IVs. More details regarding the final SNPs and the corresponding funnel plots and forest plots are summarized in Additional file 1.

## Discussion

Several hypotheses have been postulated to explain the pathogenesis of MG; these include antibodies against acetylcholine receptors (AchR), the role of CD4 + T cells in the pathogenesis of MG, and the effects of CD4 + T cell subtypes and cytokines in MG and experimental autoimmune yasthenia gravis [32–34]. Additionally, other autoantibodies such as anti-musk antibodies are observed in MG patients who lack anti-AchR antibodies [35]. It is worth noting that there is growing attention to the role of intestinal flora in the disease. Changes in either a single microbial species or the global commensal communities could play a therapeutic role in autoimmune diseases by altering the balance between pathogenic and protective immune responses [36]. Improvement in this regard has been observed in cases of rheumatoid arthritis as well as inflammatory bowel disease [37, 38]. Our study highlights the effects of alterations in gut microbiota on MG, as well as the potential for MG to cause changes in specific gut microbiota levels. Currently, this is the first two-sample bidirectional MR study to demonstrate the association between the gut microbiota and MG. Specifically, Clostridiaceae1, Defluviitaleaceae, Enterobacteriaceae, and an unknown genus were observed as having a negative relationship, while Lachnoclostridium was identified as a potential risk factor for MG. Additionally, reverse analysis revealed a negative correlation between the presence of MG and the level of Barnesiella.

### Effect of gut microbiota on MG

Our research indicates that multiple bacteria serve as inhibitory factors in the onset of MG, and the specific mechanisms may be diverse.

Foxp3 + CD4 + Treg cells play a critical role in maintaining self-tolerance and immune homeostasis by regulating the production of pathogenic antibodies through the modulation of the quantity of autoreactive T cells and the suppression of the activity of autoreactive B cells, thereby reducing the severity and progression of diseases[39]. The frequency of Foxp3 + CD4 + Treg cells in peripheral blood lymphocytes of MG patients is significantly insufficient [12]. Therefore, the abundance of Foxp3 + CD4 + Treg cells is vital for preventing and treating MG, and it has become the main focus of current research on the pathogenesis of MG. Studies have shown that gut microbiota, especially Clostridium, can affect the number of Foxp3 + CD4 + Treg cells and the surface T cell receptor (TCR). The TCR on Foxp3 + CD4 + Treg cells can recognize commensal bacterial subsets, inducing naive CD4 + T cells to differentiate into antigen-specific Foxp3 + CD4 + Treg cells, and within this way, increase their numbers. An example that highlights this is Clostridium. The strains of Clostridium are able to colonize the mucous layer that is in close proximity to the epithelial, increasing the expression of 2,3-dioxygenase and TGF-β1 [40, 41]. These changes may promote the differentiation of immature T cells, resulting in the formation of Foxp3 + CD4 + Treg cells. This biological mechanism provides a protective effect, which has been observed in germ-free mice and in the colon of humans [42, 43]. The proliferator-activated receptor (PPARγ) participates in regulating the proliferation and differentiation of immune cells and can increase the number of Foxp3 + CD4 + Treg cells through inducing differentiation [44]. Streptococcus has been proven to regulate PPARγ and its ligand 15d-PGJ2 by activating PPARγ through inhibiting certain pathways or immune cell functions [45]. Its efficacy has been confirmed in treating other immune diseases such as rheumatoid arthritis and inflammatory bowel disease [46, 47]. These findings highlight the fact that gut microbiota can affect transcriptional regulation through factors such as PPARγ, thus resulting in a tightly coordinated balance of immune system reactions.

Short-chain fatty acids (SCFAs) are non-nutritional substances produced by gut microbiota, with significant physiological regulatory functions. They provide some of the energy needed by the human body, protect the intestinal mucosal barrier, inhibit intestinal inflammation, and regulate immune responses [48, 49]. SCFAs are another metabolite that can regulate Foxp3 + CD4 + Treg cells. They have a profound influence on T cells and directly regulate the differentiation of T cells into Foxp3 + CD4 + Treg cells [43, 50]. Therefore, the gut microbiota may increase the number of Foxp3 + CD4 + Treg cells by increasing the microbial metabolites (SCFAs), indirectly exerting a protective effect.

Adjusting the gut microbiota to increase the number of Foxp3 + CD4 + Treg cells may become a new strategy for treating MG. The use of probiotics to regulate the course of MG has already been validated in animal models, with a mixture of five probiotic strains (Streptococcus thermophilus, Lactobacillus reuteri, Bifidobacterium bifidum, Lactobacillus acidophilus, and Lactobacillus casei) observed to suppress pro-inflammatory lymphocyte reactions and reduce AchR antibody levels in MG model rats, and this effect is also achieved by increasing the number of Foxp3 + CD4 + Treg cells. However, the specific strains of probiotics applicable to the human body, as well as the required dosage for maximal efficacy against different types of MG, need further investigation. Our study did not show positive results for protective bacteria mentioned above, namely Clostridium and Streptococcus, which may be due to the small sample size. However, the protective bacteria with suggestive results obtained in this study, such as Clostridiaceae1, Defluviitaleaceae, Enterobacteriaceae, and an unknown type of bacteria, broaden the scope of bacteria to be studied for future research.

In previous animal experiments, it was demonstrated that transplanting the gut microbiota of mice with MG into germ-free mice can cause movement impairments, yet the specific bacterial strain(s) responsible remain unidentified. This study represents the first evidence that Lachnoclostridium is a risk factor for the development of MG. Lachnoclostridium is an important bacteria that produces SCFAs and can exhibit anti-inflammatory effects in the body [51, 52]. While previous research has shown that SCFAs have a beneficial effect on foxp3 + CD4 + Tregs, the association of Lachnoclostridium with MG is paradoxical. The reasons for this phenomenon are complex. On the one hand, SCFAs do not always have a neuroprotective effect. For example, oral administration of SCFAs can exacerbate movement impairments in mouse models that overexpress alpha-synuclein [53], suggesting that SCFAs may produce different effects in diverse pathological contexts. On the other hand, the 16S rRNA gene sequencing of Lachnoclostridium was performed at the genus level, it cannot be determined whether specific strains or species of bacteria are associated with this inconsistent result. In addition, fecal metabolites are currently considered an external manifestation of the function of the gut microbiota [2]. Besides SCFA aforementioned, studies have demonstrated a significant correlation between the microbiota of the gut and a range of metabolic biomarkers, such as valine, leucine, xanthine, cytosine, naphthalene, and catechol [2]. These metabolic products may support the view that gut microbiota disorders are related to MG, as gut microbiota may affect the occurrence of MG through metabolic pathways such as amino acids, nucleotides, and microbial metabolism[54, 55]. This is a potential new supplement to the previously proposed antibody-mediated mechanism for MG pathogenesis. However, it should be acknowledged that it is still unclear whether Lachnoclostridium, which was identified in this study, participates in these metabolic pathways and how it contributes to the pathogenesis of MG- a point that warrants further research.

### Effect of MG on gut microbiota

In the reverse MR analysis, it was discovered that MG led to a decrease in Barnesiella. Previous studies have shown that MG can alter the relative abundance of bacterial groups in the gut microbiota. Specifically, it results in a decrease in Clostridium [56]. In contrast, this study only found a negative correlation between MG and Barnesiella, with no observed correlation with Clostridium. According to a case–control study, bacterial diversity and abundance were observed to be lower in the MG group than the healthy control group [12]. As such, the microbial diversity index may serve as a new clinical tool for evaluating the severity of MG [2]. Additionally, changes in gut microbiota that occur in MG patients are slightly associated with some clinical parameters. Combining gut microbiota with intestinal metabolites can help distinguish MG subjects from healthy controls [2]. Given the current lack of detailed data on Barnesiella, it is difficult to assess the efficacy of its level changes in evaluating diseases. Nevertheless, it is important to note that the findings of this study may offer important insights for future research.

## Limitations

There are several limitations to our study that need to be considered. Firstly, the sample population we analyzed only comprised European individuals. Consequently, the extent to which findings can be generalized to non-European populations is unclear. Secondly, due to dataset constraints, it was not possible to explore the causal relationship between gut microbiota and MG at the genus level. Thirdly, the associations we identified in our study, after multiple comparisons, were only suggestive of causal relationships, not certain. Finally, the summary statistics we used instead of raw data made it challenging to carry out subgroup analyses for MG. Therefore, in the future, more comprehensive data is needed to confirm our findings.

## Conclusion

Our research yielded evidence of a causality connection in both directions between gut microbiota and MG. We identified specific types of microbes associated with MG, which offers a fresh window into the pathogenesis of this disease and the possibility of developing treatment strategies. Nonetheless, more studies, both basic and clinical, are necessary to elucidate the precise role and therapeutic potential of the gut microbiota in the pathogenesis of MG.

### Supplementary Information

**Additional file 1. **Original data used in this study, as well as the results of Mendelian randomization analysis, heterogeneity and pleiotropy tests.

## Data Availability

All data generated or analyzed during this study are included in this published article and its Additional files.

## Abbreviations

GWAS: Genome-wide association studies
IVW: Inverse variance weighted
IVs: Instrumental variables
MG: Myasthenia gravis
MR: Mendelian randomization
PPARγ: Proliferator-activated receptor
SCFAs: Short-chain fatty acids
SNPs: Single nucleotide polymorphisms
TCR: T cell receptor
